# CD271 is a molecular switch with divergent roles in melanoma and melanocyte development

**DOI:** 10.1038/s41598-019-42773-y

**Published:** 2019-05-22

**Authors:** Fabian V. Filipp, Chen Li, Alexander D. Boiko

**Affiliations:** 1 Cancer Systems Biology, Institute of Computational Biology, Helmholtz Zentrum München, Ingolstädter Landstraße 1, D-85764 München, Germany; 2 School of Life Sciences Weihenstephan, Technical University München, Maximus-von-Imhof-Forum 3, D-85354 Freising, Germany; 3 Systems Biology and Cancer Metabolism, Program for Quantitative Systems Biology, University of California Merced, 5200 North Lake Road, Merced, CA 95343 USA; 4 Sue and Bill Gross Research Stem Cell Center, University of California Irvine, 845 Health Sciences Road, Irvine, CA 92697 USA; 5 Department of Molecular Biology and Biochemistry, University of California Irvine, 845 Health Sciences Road, Irvine, CA 92697 USA

**Keywords:** Melanoma, Regulatory networks

## Abstract

Dysregulation of signaling networks controlling self-renewal and migration of developmental cell lineages is closely linked to the proliferative and invasive properties of tumors. Identification of such signaling pathways and their critical regulators is vital for successful design of effective targeted therapies against neoplastic tissue growth. The neurotrophin receptor (CD271/NGFR/p75NTR) is a key regulator of the melanocytic cell lineage through its ability to mediate cell growth, survival, and differentiation. Using clinical melanoma samples, normal melanocytes and global gene expression profiling we have investigated the role of CD271 in rewiring signal transduction networks of melanoma cells during neoplastic transformation. Our analysis demonstrates that depending on the cell fate of tumor initiation vs normal development, elevated levels of CD271 can serve as a switch between proliferation/survival and differentiation/cell death. Two divergent arms of neurotrophin signaling hold the balance between positive regulators of tumor growth controlled by E2F, MYC, SREBP1 and AKT3 pathways on the one hand, and differentiation, senescence, and apoptosis controlled by TRAF6/IRAK-dependent activation of AP1 and TP53 mediated processes on the other hand. A molecular network map revealed in this study uncovers CD271 as a context-specific molecular switch between normal development and malignant transformation.

## Introduction

Melanoma represents one of the most aggressive types of cancer due to its high proliferative and metastatic potential^1^. Multiple studies have indicated that melanoma progression partially recapitulates a developmental program of normal melanocytic lineage, by hijacking signaling pathways active in stem/progenitor cell populations during normal development^2–5^. At the embryonic stages, melanocytes and their precursors, melanoblasts, are derived from a multipotent stem cell population, neural crest^6^. This highly proliferative and migratory cell population can be defined by the expression of the nerve growth factor receptor, CD271(NGFR/p75NTR)^7,8^.

Our group and others have previously demonstrated that within human melanomas, cells expressing neural crest stem cell marker, CD271, represent the most aggressive tumor-and metastasis-initiating cell population^9–11^. Using surgically removed human tumors combined with an advanced techniques of cell isolation these studies demonstrated that CD271^+^ melanoma cells have increased tumor-initiating capacity, self-renew and can serially transplant the disease *in-vivo* recapitulating original patient tumor morphology and heterogeneity giving rise to CD271^+^ and CD271^−^ cells^9,10^. Despite an ongoing debate whether frequency of CD271^+^ human tumor-initiating cells is over- or under-estimated as a result of modifications in the mouse xenotransplantation protocols, including the use of high-protein matrigel^12,13^, their true physiological frequency in human patients cannot be determined due to the fact that it would require isogenic transplantations, which are impossible to perform. Nonetheless, the clinical value of melanoma-initiating cells, namely characterization of their phenotypic and molecular properties bears significant impact on the development of targeted anti-melanoma therapeutic regimens^14–21^.

Since their identification, melanoma-initiating cells and high levels of CD271 expression have been associated with metastatic progression, enhanced survival, resistance to the chemotherapeutic agents, including MAPK inhibitors, and evasion of the immune system, through de-differentiation and downregulation of T-Cell activating antigens^9,10,14–16,18–20,22–25^. Antibody-mediated targeting of CD271^+^ melanoma cells has recently been shown to synergize with the activation of an innate immune response via CD47 blockade and dramatically reduce tumor growth, as well as, the lymph node and distant organ metastases in mice xenotransplanted with patient derived melanomas^9^. Downregulation of CD271 using shRNA mediated gene knockdown abolishes tumorigenic growth of melanoma cells *in-vivo*, reduces their migratory properties and predisposes them to the DNA damaging, apoptosis inducing, stimuli^11^. Tumorigenic stem cell properties of CD271 signaling had also been demonstrated for head and neck cancers, where its knockdown led to the defects in cell proliferation and tumor regression^26^.

Neurotrophins (nerve growth factors) mediate growth, death, survival, and differentiation of the cells when they bind to their cognate target receptors^27^. They are recognized by two classes of receptors, CD271 and receptor tyrosine kinase (RTK/TRK) members, both of which belong to the large tumor necrosis factor receptor superfamily^28^. These receptors transmit intracellular signals through several canonical pathways, including the mitogen activated protein kinase (MAPK) and phosphatidylinositol-3 kinase (PI3K)/AKT signaling pathways^29^. Conversely, CD271 has the ability to facilitate a broad functional spectrum including opposing functions of cell death and survival in its capacity as signaling receptor for stem cell or inhibitory components^30–32^. Thus, the physiological consequences of CD271 depend on the cellular context of its signaling^33^.

Unraveling heterogeneity of CD271 signaling to discover new subpopulations of cells within the tumor has been fundamental to many advances in cancer biology, including identification of tumor-initiating subsets and cells resisting immune-therapeutic regimens^9,18–20^. In this systems biology study, using clinical melanoma samples, fluorescent-activated cell sorting (FACS) in combination with global transcriptome profiling, we report distinct signaling pathways connected to CD271 expression in melanoma and melanocytes. CD271^+^ cells derived from human melanoma patients in comparison to CD271^−^ counterparts and CD271^+^ cells derived from normal melanocytic lineages elucidate a contrasting network response during normal development and malignant transformation.

## Results

### Generation of patient-derived CD271^+^ vs CD271^−^ transcriptome profiles

**Figure 1:**
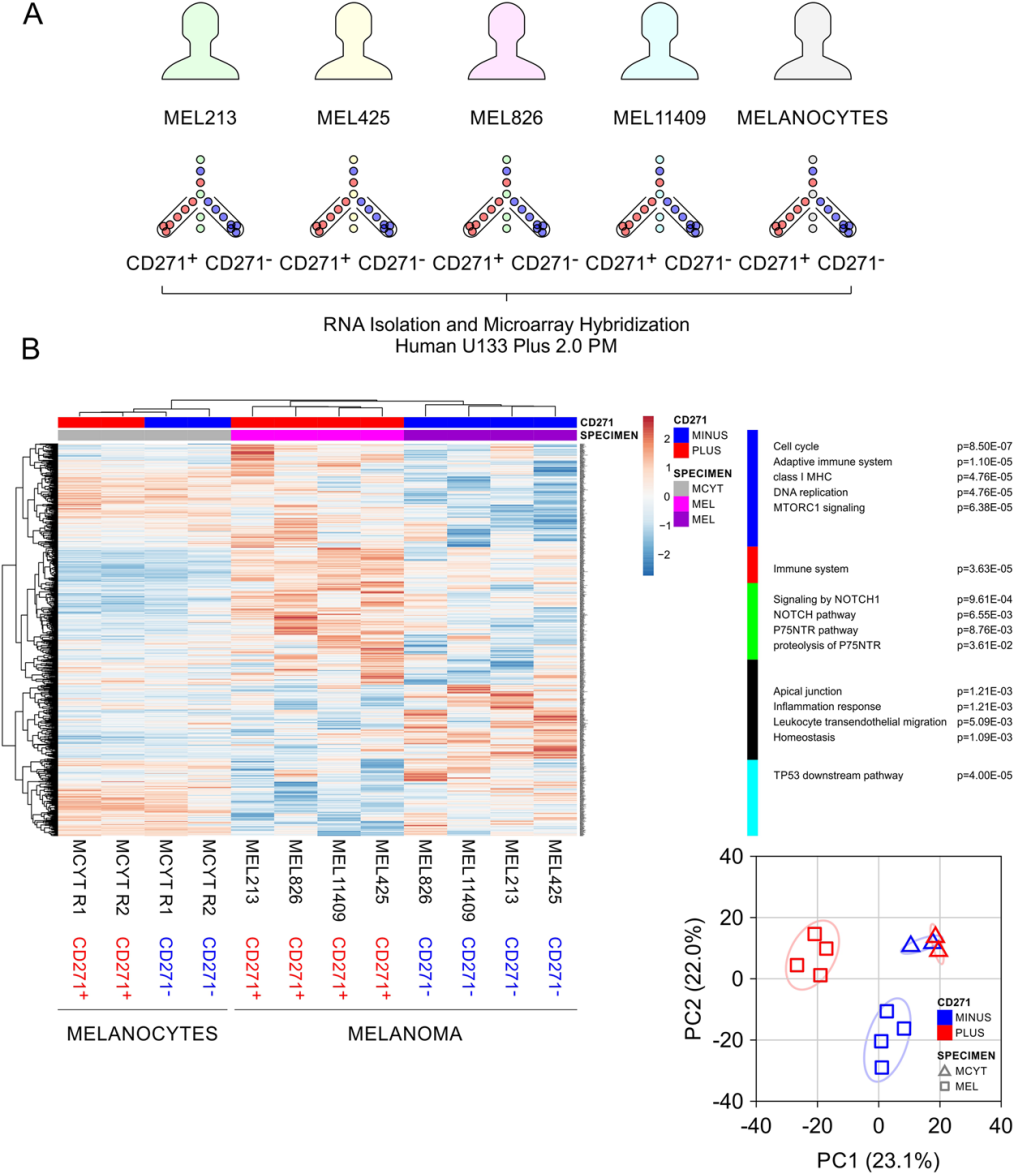
CD271 expression has a strong impact on melanoma transcriptome and has distinct effect in melanocytes. (**A**) Experimental design of patient-derived transcriptomic profiles of CD271^+^ and CD271^−^ melanoma cells in comparison with profiles of healthy donor melanocytes. All cell populations were sorted by flow cytometry and subjected to transcriptomic profiling. (**B**) Heat map of gene expression profiles and indication of CD271 status in tumor cells (pink and purple) vs normal melanocytes (grey). Selfrenewal, cell cycle and cell survival pathways are significantly overrepresented with *p* values below 0.05 in CD271^+^ melanoma-initiating cells vs CD271^−^ cells and normal melanocytes. Principal component analysis (PCA) reveals separation of expression profiles.

We previously used clinical melanoma tumor specimens to classify CD271^+^ cell populations as tumor-initiating^9^. Isolation of these cells to the highest degree of purity provided unique opportunity to compare transcriptional profiles across multiple patients, between CD271^+^ and CD271^−^ populations. We utilized flow cytometry in combination with lineage- and CD271-specific monoclonal antibodies to purify distinct CD271^+^ and CD271^−^ tumor cell subsets from surgically removed, human tumor tissue specimens, described in detail^9^ (Fig. 1A). In addition, we used the same approach to purify CD271^+^ and CD271^−^ cell population from epidermal melanocytes separately isolated from healthy human skin tissue. All CD271^+^ and CD271^−^ cell populations were then subjected to RNA extraction, transcriptomic profiling, and systems biology analysis.

Clustering based on Pearson correlation, principal component analysis (PCA), and heatmap visualization provided a global overview of patient-derived CD271^+^ vs CD271^−^ transcriptome profiles of melanomas and melanocytes. In the column dimension of the clustering, melanomas and melanocytes specimens were segregated based on CD271 status (Fig. 1B). The row dimension provided first insight into processes that were differently expressed in melanoma-initiating CD271^+^ cells vs CD271^−^ cells from matching tumors that were unable to initiate tumor growth *in-vivo* or had a much lower efficiency^9^. In addition, separate clusters of CD271^+^ and CD271^−^ melanocytes provided second dimension of comparison revealing specific signaling pathways unique to tumor-initiating CD271^+^ melanoma cells (Fig. 1B). The first branches of the row tree included cell cycle progression, pathways of neurotrophin and NOTCH signaling, cell survival and immune responses. Gene members of such pathways were in general higher expressed in the CD271^+^ melanoma-initiating cells compared to CD271^−^ melanoma cells and CD271^+^ melanocytes. The next branches of the row tree included cell-cell contacts, tissue homeostasis, and TP53 mediated singing networks of cell cycle arrest and apoptosis. Importantly, these pathways, typical for tissue differentiation, were downregulated in CD271^+^ melanoma cells, but were upregulated in CD271^−^ counterparts and had the strongest expression in the CD271^+^ normal melanocytes (Fig. 1B).

The patient-derived tumor specimens in this study represented a considerable amount of heterogeneity including different site of diagnosis, and the status of BRAF activation (Supplementary Table 1). Nonetheless, PCA based on the cell surface CD271 status, separated melanoma and melanocyte specimens into two clusters with 50% data representation in the first two principal components (PC1 = 28% and PC2 = 22%) (Fig. 1B). The principal components showed that the average perturbation of the CD271^+^ melanoma-initiating cells is in the opposite direction and of greater magnitude compared to the CD271^+^ melanocytes. CD271 expression had a strong impact on melanoma transcriptomes yet unsupervised clustering and PCA showed distinct effects in melanocytes (Fig. 1B).

**Figure 2:**
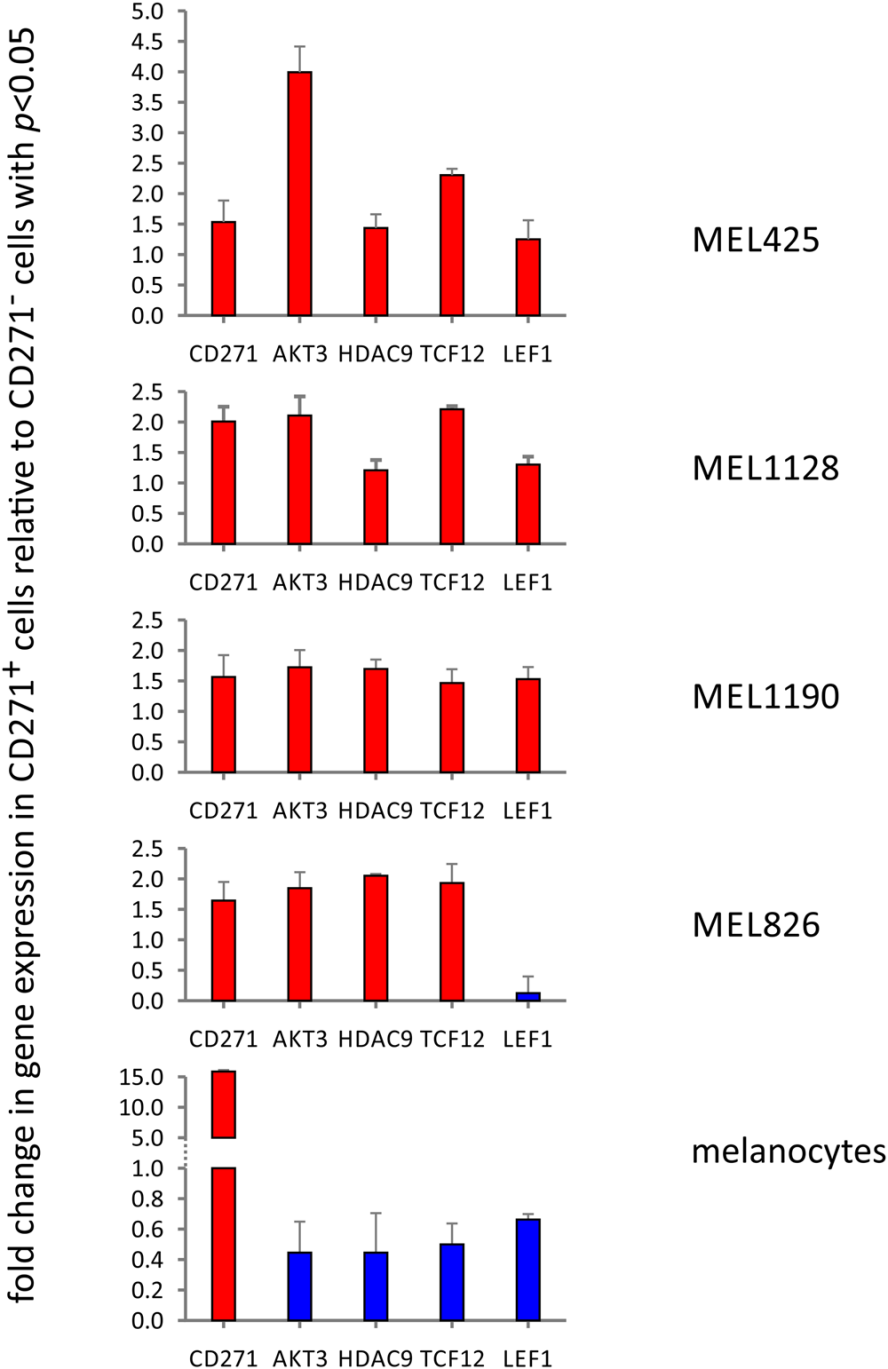
Transcriptional validation of differentially expressed genes by qRT-PCR based on CD271 status and normalization against 18 s RNA using the ΔCT method. The fold change for a transcript of interest was determined by taking the ratio of normalized gene expression between CD271^+^ and CD271^−^ cell populations for each patient sample. In brief, differential gene expression was determined by calculating the fold change of any gene of interest (GOI) = 2^−ΔΔCT^, where ΔCT(GOI) = (CT(GOI) − CT(18 s) and ΔΔCT(GOI) = ΔCT(GOI-condition A) − ΔCT(GOI condition B) with CD271^+^ and CD271^−^ for condition A and B, respectively.

Next, we used qRT-PCR and gene-specific primers (Supplementary Table 2) and FACS sorted CD271^+^/CD271^−^ melanoma cell populations, as well as, CD271^+^/CD271^−^ normal melanocytes, to confirm specific clusters of CD271 mediated gene expression. We validated top hits of representative genes involved in self-renewal, cell survival, and epigenetic rewiring. As a positive control we also measured gene expression of *CD271* in the same cell populations. Thus, *AKT3*, *HDAC9*, *TCF12*, and *LEF1* were differentially expressed in the melanoma specimens, tracking with CD271 enrichment (Fig. 2). In contrast, melanocytes reversed transcription levels of such markers, despite elevated levels of CD271.

Taken together, the CD271 effect between CD271^+^ vs CD271^−^ cells created distinct clusters of transcripts that were functionally connected by cellular pathways. Nevertheless, given an underlying cellular fate of oncogenic program vs normal development, an overarching theme of opposing signaling branches separated CD271^+^ melanoma and melanocyte enriched cells with transcriptional responses in opposite directions.

### CD271 positively regulates gene networks associated with melanoma progression

**Figure 3:**
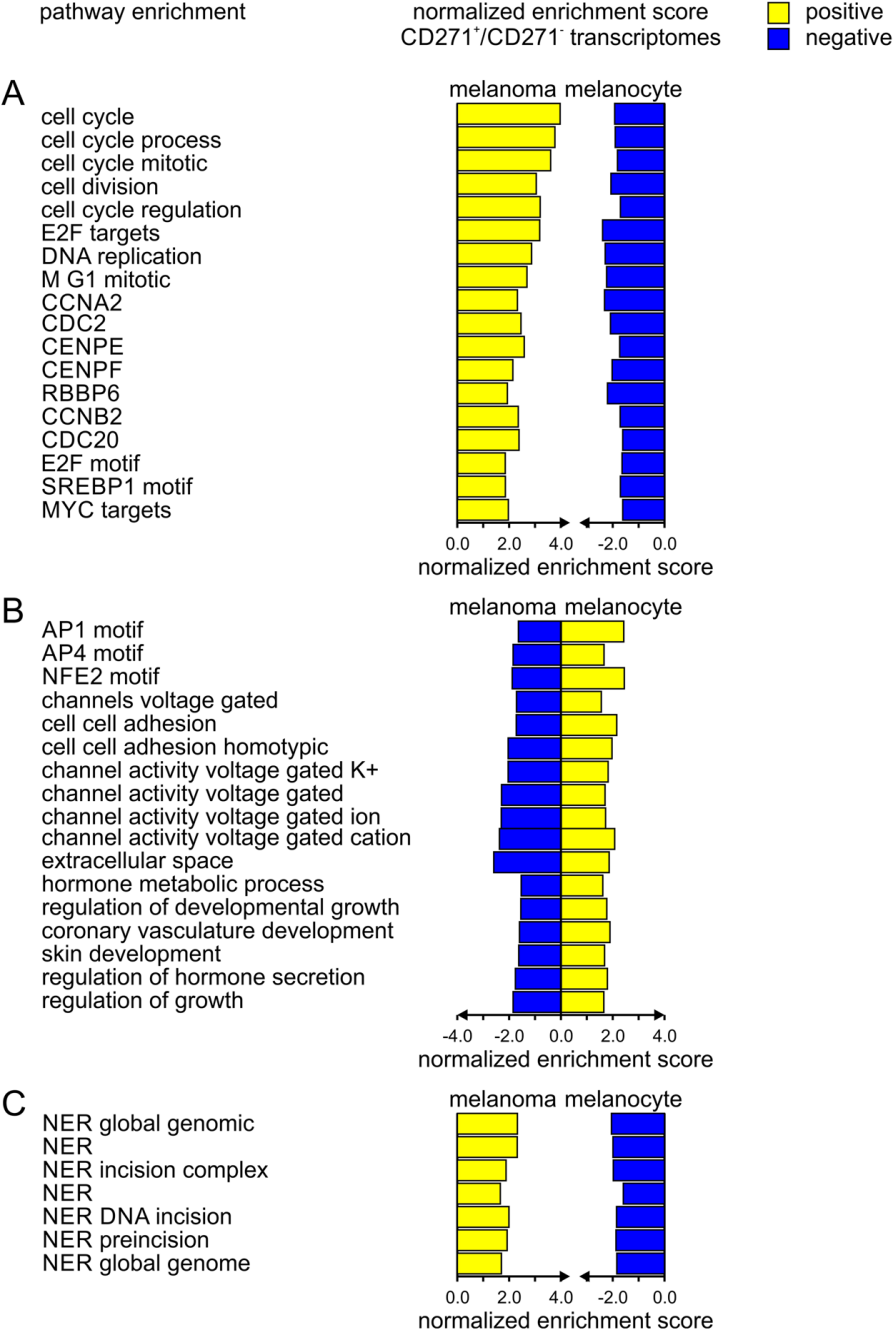
Stimulation of the cell cycle, DNA repair and cell survival in CD271^+^ melanoma cells, in contrast to emphasis on differentiation processes in CD271^−^ cells and normal melanocytes. The ratio of CD271^+^/CD271^−^ transcriptomes reveals enrichment of CD271-associated pathways. Directional, normalized enrichment scores are color-coded (positive in yellow, negative in blue) and functionally grouped by cell type for melanoma and melanocytes for (**A**) cell cycle processes, (**B**) differentiation processes, and (**C**) nucleotide excision repair (NER).

**Figure 4:**
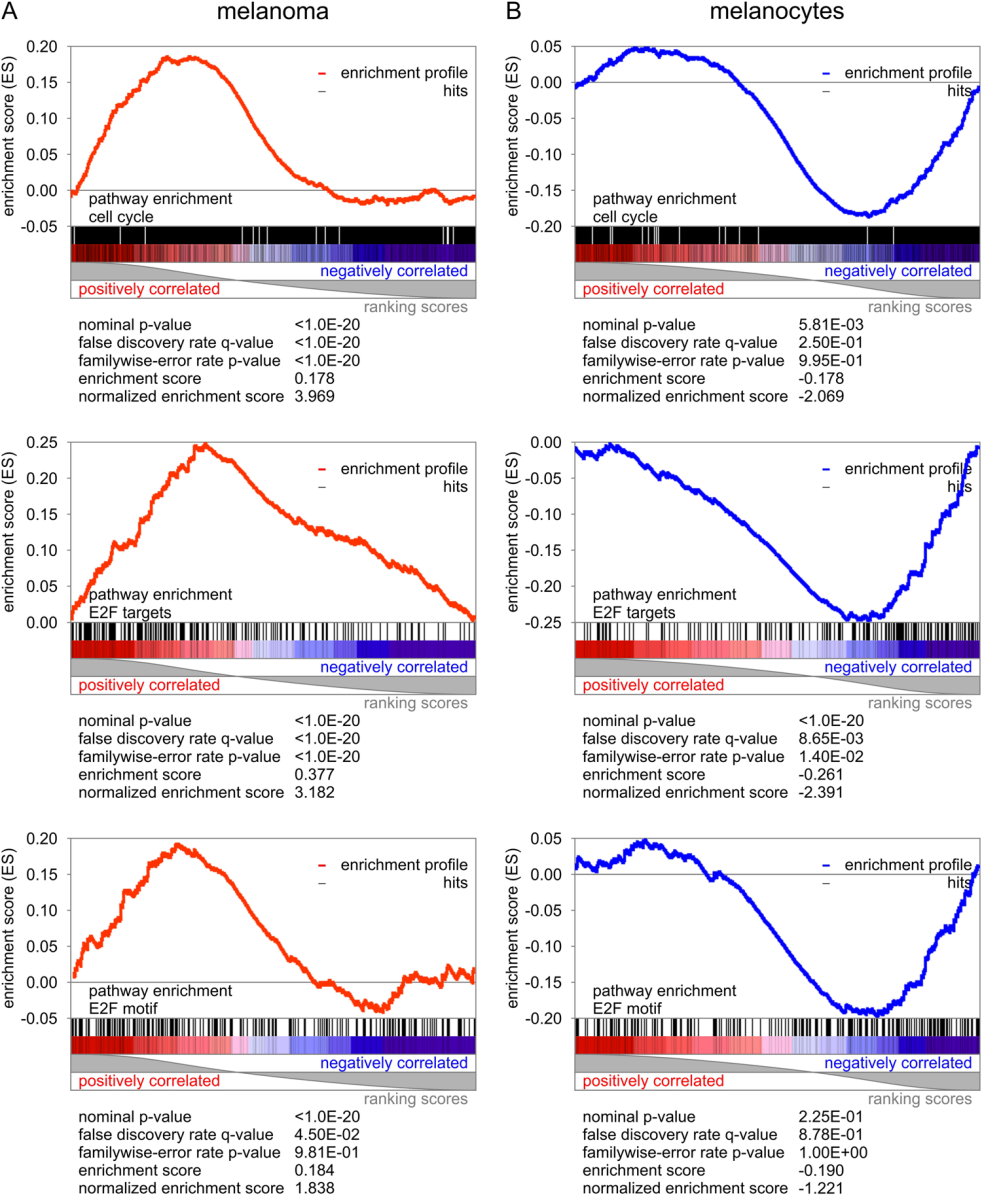
Overrepresentation of the E2F transcription factor responsive pathways and cell cycle processes in CD271^+^ melanoma cells in contrast to differentiation processes in CD271^−^ cells and normal melanocytes. Gene set enrichment analysis with statistical scores for (**A**) melanoma and (**B**) melanocytes.

Our next step was to delineate cellular pathways that were uniquely associated with CD271^+^ in comparison to CD271^−^ in melanoma and melanocyte cells. CD271 expression had a strong impact on melanoma transcriptomes yet unsupervised clustering and PCA showed distinct, opposite effects in CD271^−^ melanoma cells and melanocytes (Figs 1–3). CD271^+^ melanoma-initiating cells showed significant overrepresentation of proliferative pathways in comparison to CD271^−^ matching tumor cells, as well as, CD271^+^ melanocyte transcriptomes (Fig. 3). Key pathways of cell cycle processes, E2F effector target genes, and DNA replication were significantly enriched with *p* values below 0.05 and *q* values below 0.25. Intriguingly, the same pathways that are upregulated in melanoma in the presence of CD271 are downregulated in CD271^+^ melanocytes (Fig. 3). Transcripts associated with representatives of cell cycle regulators including cyclins (CCNA2, CCNB2, CCNE1), cyclin-dependent protein kinases (CDC2, CDC20), centromere proteins (CENPE, CENPF), retinoblastoma binding proteins (RBBP6), were all enriched in CD271^+^ melanoma-initiating cells but downregulated in CD271^−^ melanoma cells and CD271^+^ melanocytes. As a result, target genes carrying E2F sequence motifs in their promoter sites are enriched in CD271^+^ melanoma cell population (Fig. 4). Moreover, differential analysis of transcriptomic data revealed that gene nodes associated with non-canonical WNT or NOTCH signaling were significantly activated in CD271^+^ melanoma-initiating cells, but they were absent from or not activated in CD271^−^ melanoma cells and terminally differentiated melanocytes (Figs 1–3, Table 1). Specifically, we revealed strong activation of the basic helix-loop-helix transcriptional complex of TCF and LEF effector genes. In addition, selected members of NFKB and NOTCH signaling pathways (IKBKB, NOTCH2, HES1, PRKCA) were strongly overrepresented in CD271^+^ melanoma-initiating cells (Table 1).

**Table 1:** Significant overrepresentation of tumor progression associated pathways in CD271^+^ melanoma-initiating cells vs CD271^−^ melanoma cells, and vs normal melanocytes.

Pathway	Melanoma			Melanocytes		
	NES	*p* value	*q* value	NES	*p* value	*q* value
E2F targets	**3.18**	0.00E + 00	0.00E + 00	*−2.39*	0.00E + 00	8.65E − 03
MYC targets	**1.86**	4.01E − 03	4.50E − 02	*−1.70*	3.19E − 02	2.28E − 01
nucleotide excision repair	**2.31**	1.91E − 03	1.88E − 02	*−1.99*	6.07E − 03	8.04E − 02
dna replication	**2.87**	0.00E + 00	0.00E + 00	*−2.29*	0.00E + 00	1.53E − 01
mitotic m m g1 phases	**2.69**	0.00E + 00	8.50E − 04	*−2.24*	0.00E + 00	7.42E − 02
global genomic ner gg ner	**2.32**	4.12E − 03	1.72E − 02	*−2.05*	1.00E − 02	1.44E − 01
class i mhc mediated antigen processing presentation	**2.17**	4.32E − 03	3.70E − 02	*−1.90*	1.01E − 02	2.37E − 01
metabolism of rna	**2.14**	1.93E − 03	4.03E − 02	*−2.00*	8.03E − 03	1.73E − 01
mitotic prometaphase	**2.09**	0.00E + 00	5.62E − 02	*−1.89*	1.36E − 02	2.38E − 01
antigen processing ubiquitination proteasome degradation	**2.02**	2.22E − 03	7.67E − 02	*−1.86*	1.22E − 02	2.11E − 01
metabolism of mrna	**1.91**	6.56E − 03	9.71E − 02	*−2.21*	1.95E − 03	6.75E − 02
formation of incision complex in gg ner	**1.88**	1.24E − 02	9.93E − 02	*−1.98*	5.93E − 03	1.78E − 01
deadenylation dependent mrna decay	**1.69**	2.07E − 02	1.65E − 01	*−1.89*	3.95E − 03	2.20E − 01
CENPE	**2.58**	2.07E − 03	3.62E − 03	*−1.73*	2.08E − 02	2.25E − 01
CDC2	**2.46**	2.07E − 03	4.39E − 03	*−2.09*	7.81E − 03	8.91E − 02
CCNB2	**2.35**	0.00E + 00	5.86E − 03	*−1.71*	2.12E − 02	2.30E − 01
CCNA2	**2.32**	2.11E − 03	6.96E − 03	*−2.32*	0.00E + 00	5.05E − 02
SMC2L1	**2.31**	0.00E + 00	6.86E − 03	*−1.77*	1.62E − 02	2.00E − 01
HMMR	**2.31**	2.00E − 03	6.55E − 03	*−1.95*	6.13E − 03	1.21E − 01
RRM1	**2.23**	0.00E + 00	9.37E − 03	*−2.29*	4.07E − 03	4.64E − 02
GCM CHUK	**2.23**	0.00E + 00	9.75E − 03	*−1.74*	2.25E − 02	2.26E − 01
SMC4L1	**2.16**	0.00E + 00	1.45E − 02	*−2.16*	0.00E + 00	6.91E − 02
CENPF	**2.14**	0.00E + 00	1.60E − 02	*−2.02*	8.37E − 03	1.05E − 01
CKS1B	**2.14**	0.00E + 00	1.52E − 02	*−1.87*	4.00E − 03	1.53E − 01
MORF RFC4	**2.09**	0.00E + 00	1.77E − 02	*−1.82*	1.35E − 02	1.70E − 01
PCNA	**1.95**	1.39E − 02	3.60E − 02	*−2.01*	8.60E − 03	1.05E − 01
RBBP6	**1.93**	1.43E − 02	3.65E − 02	*−2.20*	0.00E + 00	5.82E − 02
TTK	**1.89**	1.15E − 02	4.36E − 02	*−1.95*	3.88E − 03	1.27E − 01
MCM4	**1.80**	1.61E − 02	6.28E − 02	*−1.80*	1.80E − 02	1.77E − 01
MKI67	**1.72**	3.09E − 02	8.16E − 02	*−1.69*	2.56E − 02	2.30E − 01
RFC4	**1.71**	2.73E − 02	8.55E − 02	*−2.08*	4.13E − 03	8.40E − 02
CKS2	**1.69**	2.44E − 02	8.81E − 02	*−2.28*	0.00E + 00	3.94E − 02
cell division	**3.05**	0.00E + 00	3.91E − 04	*−2.07*	5.81E − 03	2.50E − 01
sister chromatid segregation	**2.45**	0.00E + 00	2.02E − 02	*−2.38*	0.00E + 00	1.62E − 01
organelle fission	**2.29**	1.96E − 03	4.17E − 02	*−2.11*	1.98E − 03	2.17E − 01
nuclear chromosome segregation	**2.25**	1.97E − 03	4.75E − 02	*−2.15*	1.96E − 03	1.99E − 01
chromosome segregation	**2.23**	0.00E + 00	4.63E − 02	*−2.52*	0.00E + 00	6.91E − 02
macromolecule catabolic process	**2.02**	2.15E − 03	1.08E − 01	*−2.16*	3.85E − 03	2.11E − 01
regulation of proteasomal protein catabolic process	**1.94**	7.95E − 03	1.39E − 01	*−2.04*	4.12E − 03	2.38E − 01
regulation of proteasomal ubiquitin process	**1.76**	1.26E − 02	2.33E − 01	*−2.30*	2.01E − 03	1.84E − 01
transferase complex	**2.73**	0.00E + 00	1.49E − 03	*−2.22*	0.00E + 00	2.35E − 01
condensed chromosome centromeric region	**2.21**	0.00E + 00	3.15E − 02	*−2.10*	1.99E − 03	1.71E − 01
condensed chromosome	**2.16**	0.00E + 00	3.17E − 02	*−1.90*	9.56E − 03	2.45E − 01
catalytic complex	**2.03**	2.09E − 03	5.35E − 02	*−1.97*	0.00E + 00	2.45E − 01
nucleoplasm part	**1.91**	4.29E − 03	8.96E − 02	*−1.94*	6.24E − 03	2.49E − 01
kinetochore	**1.66**	3.30E − 02	2.00E − 01	*−1.90*	7.83E − 03	2.23E − 01
voltage gated cation channel activity	*−2.37*	0.00E + 00	1.30E − 02	**2.08**	0.00E + 00	1.74E − 01
ERB2 up.v1 dn	**3.56**	0.00E + 00	0.00E + 00	*−3.16*	0.00E + 00	0.00E + 00
MEK up.v1 dn	**2.30**	2.04E − 03	1.74E − 02	*−1.65*	4.59E − 02	2.25E − 01
EGFR up.v1 dn	**1.90**	6.01E − 03	6.77E − 02	*−1.63*	4.11E − 02	2.17E − 01
VEGF a up.v1 dn	**1.61**	3.01E − 02	1.77E − 01	*−1.66*	3.09E − 02	2.36E − 01
unstim vs curdlan highdose stim dc up	**3.27**	0.00E + 00	3.52E − 04	*−2.19*	0.00E + 00	1.65E − 01
day6 vs day10 traf6ko eff cd8 tcell up	**3.02**	0.00E + 00	5.32E − 04	*−2.90*	0.00E + 00	1.12E − 02
day1 vs day7 yf17d vaccine pbmc dn	**2.98**	0.00E + 00	4.73E − 04	*−2.33*	0.00E + 00	1.07E − 01
nstim vs mcsf treated monocyte day7 up	**2.91**	0.00E + 00	5.55E − 04	*−2.05*	3.87E − 03	2.24E − 01
ctrl vs anti igm stim bcell 12 h up	**2.72**	0.00E + 00	2.18E − 03	*−2.16*	0.00E + 00	1.90E − 01
ctrl vs anti igm stim bcell 2 h up	**2.67**	0.00E + 00	2.38E − 03	*−2.33*	0.00E + 00	1.03E − 01
day15 effector vs day30 exhausted cd8 tcell lcmv dn	**2.48**	0.00E + 00	6.34E − 03	*−2.35*	2.02E − 03	1.06E − 01
ctrl vs tgfbeta1 il6 il23a cd4 tcell up	**2.41**	0.00E + 00	8.57E − 03	*−2.69*	0.00E + 00	3.05E − 02
naive vs day4.5 eff cd8 tcell dn	**2.32**	0.00E + 00	1.23E − 02	*−2.07*	0.00E + 00	2.27E − 01
naive vs klrg1high eff cd8 tcell dn	**2.29**	0.00E + 00	1.37E − 02	*−2.27*	0.00E + 00	1.33E − 01
n vs fat treg up	**2.23**	2.02E − 03	1.78E − 02	*−2.42*	0.00E + 00	9.79E − 02
a2ar ko vs a2ar agonist treated treg dn	**2.21**	2.11E − 03	1.97E − 02	*−2.19*	1.97E − 03	1.71E − 01
day6 vs day10 eff cd8 tcell up	**2.15**	2.03E − 03	2.53E − 02	*−3.00*	0.00E + 00	7.10E − 03
iver dc vs pln dc activated allogenic tcell dn	**2.01**	6.05E − 03	4.36E − 02	*−2.15*	1.93E − 03	1.78E − 01
gfb and il4 vs tgfb and il12 treated act cd4 tcell 6 h dn	**2.00**	8.11E − 03	4.51E − 02	*−2.07*	1.96E − 03	2.32E − 01
tconv vs foxp3 ko induced treg dn	**1.99**	6.07E − 03	4.65E − 02	*−2.39*	0.00E + 00	1.07E − 01
teff vs tconv day5 in culture up	**1.96**	1.26E − 02	5.12E − 02	*−2.22*	0.00E + 00	1.56E − 01
ctrl vs cpg 1 h bmdc up	**1.95**	8.46E − 03	5.54E − 02	*−2.16*	0.00E + 00	1.84E − 01
tconv vs treg 24 h tnf stim up	**1.89**	1.19E − 02	6.61E − 02	*−2.53*	0.00E + 00	5.21E − 02
nstim vs mcsf treated monocyte day3 up	**1.83**	1.38E − 02	8.20E − 02	*−2.20*	2.02E − 03	1.76E − 01
wt vs sap1a ko dp thymocytes up	**1.82**	1.22E − 02	8.50E − 02	*−2.07*	5.78E − 03	2.27E − 01
2 h vs 12 h anti igm stim bcell up	**1.74**	2.69E − 02	1.09E − 01	*−2.28*	4.12E − 03	1.30E − 01
ctrl vs tgfbeta3 il6 il23a cd4 tcell up	**1.74**	3.93E − 02	1.10E − 01	*−2.37*	0.00E + 00	1.10E − 01
spleen c57bl6 vs 4t1 tumor balbc monocytes dn	**1.73**	2.60E − 02	1.15E − 01	*−2.47*	0.00E + 00	7.72E − 02
laiv vs tiv flu vaccine day7 pdc up	**1.70**	3.05E − 02	1.26E − 01	*−2.05*	5.76E − 03	2.23E − 01
wt vs stat6 ko macrophage dn	**1.69**	1.90E − 02	1.27E − 01	*−2.15*	1.88E − 03	1.79E − 01
untreated vs tgfb il6 treated cd4 tcell up	**1.65**	3.41E − 02	1.47E − 01	*−2.58*	0.00E + 00	4.25E − 02
wt vs ikaros ko granulocyte monocyte progenitor up	**1.64**	3.33E − 02	1.50E − 01	*−2.30*	0.00E + 00	1.17E − 01
STAT5 ab knockin vs wt tcell il2 treated 6 h dn	**1.63**	2.54E − 02	1.50E − 01	*−2.19*	0.00E + 00	1.68E − 01
protein secretion	**3.01**	0.00E + 00	0.00E + 00	**2.01**	3.88E − 03	3.28E − 02
fatty acid metabolism	**1.89**	7.69E − 03	4.76E − 02	**2.02**	0.00E + 00	3.91E − 02
morf AP2m1	**2.57**	0.00E + 00	3.30E − 03	**2.28**	0.00E + 00	1.51E − 01

The enrichment study includes normalization of the enrichment score accounting for size of each gene set, yielding the normalized enrichment score (NES). In addition to the calculated probability during hypothesis testing (*p* value), there is adjustment for multiple hypotheses testing by controlling the proportion of false positives by calculating the false discovery rate (*q* value) corresponding to each NES, and by comparing tails of the observed and the null distribution for the NES. Scores are highlighted in bold and italic for positive and negatives NES values, respectively.

Overall, CD271^+^ melanoma cells were associated with a highly proliferative and mitotic state as compared to CD271^−^ cells from the matching tumors. In contrast, CD271^+^ cells derived from normal melanocytes were enriched for the pathways and processes driving differentiation (Figs 3, 4; Table 1).

### Impact of CD271 on cell survival and DNA repair processes

**Figure 5:**
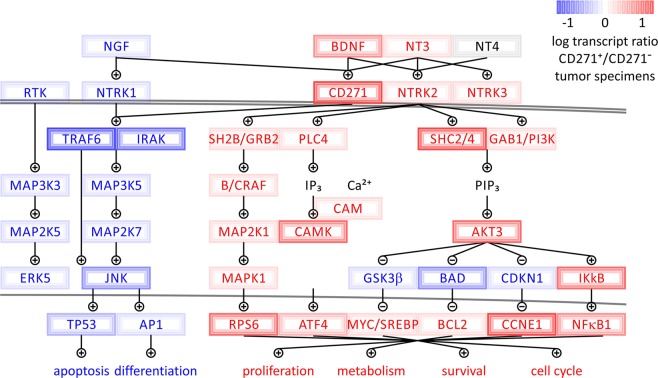
Switch in the neurotrophin pathway depends on the expression of the nerve growth factor receptor (CD271/NGFR/p75NTR). Gene expression levels plotted onto map of the neurotrophin pathway in CD271^+^ melanoma-initiating cells. Two divergent arms of neurotrophin signaling balance between self-renewing, cell survival and cell cycle signals on the one hand, and developmental, differentiation and apoptosis triggers on the other hand.

Gene expression analysis identified activation of AKT3, but not AKT1 or AKT2, as a major cell survival node upregulated in CD271^+^ melanoma-initiating cells. Using system pathway profiling analysis we reveal the molecular network model (Fig. 5), which illustrates how different neurotrophin receptor-dependent adaptor proteins, including GRB2, SHC, and GAB1 connect activation of CD271 to the upregulation of AKT3 in CD271^+^ melanoma-initiating cells. In addition to GAB1 and SHC-dependent PI3K signaling, tyrosine or serine/threonine kinases have been shown to activate AKT directly, in response to growth factors, inflammation or DNA damage. These can function even when PI3K activity is inhibited. AKT can also be activated in response to increases in cellular Ca^2+^ concentration, via Ca^2+^/Calmodulin-dependent protein kinase II (CAMKII). All of the above molecular components were transcriptionally upregulated in CD271^+^ melanoma-initiating cells, while being absent or downregulated in CD271^−^ counterparts and CD271^+^ melanocytes (Table 1, Figs 1B, 5).

The differential analysis filtered for processes driven by CD271 in tumor-initiating cells also revealed enrichment for the genes participating in nucleotide excision repair (NER, including damage recognition and repair factor XPC, DNA repair associated BRCA2, UBE2I, COPS7A, POLD3, USP45, RFC5) (Fig. 3C). Activation of NER pathways bears special significance to the tumor-initiating cells with stem-like properties, which have to limit the extent of genomic damage in order to survive for prolonged periods of time.

### Differential impact of CD271 on melanocyte development as compared to melanomagenesis

Developmental pathways that are important to direct differentiation within the melanocytic lineage were enriched in CD271^+^ melanocytes but are inhibited in CD271^+^ melanoma-initiating cells. Thus, signal transduction networks associated with skin development, metabolic hormone processes, cell adhesion, and ion channel activity are significantly enriched in CD271^+^ melanocytes with *p* values below 0.05 and *q* values below 0.25. The same pathways that are upregulated in melanocytes and important maintenance and differentiated tissue are downregulated in melanoma in the presence of CD271 (Figs 3, 4). In addition, the neurotrophin signaling showed distinct rewiring, where CD271 expression correlated with switched activity of JNK and TRAF6 pathways in melanoma and melanocytes (Fig. 5). Interestingly, our analysis established that CD271^+^ melanoma cells had similar gene expression profiles to *TRAF6* knockout cell lines^34^. Neurons with *TRAF6* associated with CD271 operate JNK-dependent apoptosis to guide cerebellar development^35^. In the same fashion, CD271 signaling in melanoma does not rely on TRAF6, which is significantly downregulated (Fig. 5). In contrast, when the same CD271 receptor is enriched and active in melanocytic lineages during physiologic conditions, it stimulates terminal differentiation leading to non-proliferative states and expression of pigment associated melanocytic markers (including keratins KRT1, KRT9, KRT16, KRT25, KRT71, collagen type I alpha 2 chain COL1A2, differentiation transcription factors FOXE1, HOXC13, SRF, FOSL2, growth factors TGFB2, FGF10, SOS1, integrins ITGA2, ITGB4, CD109, and WNT16) (Table 1).

## Discussion

In the present study, we demonstrate that depending on the state of the tissue homeostasis elevated levels of the neurotrophin receptor, CD271, can serve as a cell proliferative/survival switch for melanoma-initiating cells or as a differentiation switch for developing melanocytes. In melanoma and melanocytes, CD271 was significantly associated with similar networks yet opposing roles. Comprehensive analysis of highly purified CD271^+^ and CD271^−^ cell populations from malignant melanomas and normal melanocytes revealed two divergent arms of neurotrophin signaling, which balance between proliferative cell cycle signals, survival and epigenetic rewiring on one hand, and apoptosis, senescence, and developmental triggers on the other hand. Thus, significant pathway enrichment connected CD271 in melanoma with gene targets of E2F, MYC, SREBP1, and PI3-kinase signaling (Figs 1, 3, 4, Table 1). At the same time, differentiation and cell death triggers including TRAF6-dependent JUN-kinase activation of the AP1 complex and TP53 was suppressed (Fig. 5).

The systems biology analysis performed in this study revealed that upregulation of self-renewing genes in CD271^+^ melanoma cells occurs in response to the activation of E2F, MYC, and SREBF1 promoter-containing elements. Target genes containing these response elements in their promoters (summarized in Table 1) are known to hold critical roles in cell cycle progression and DNA replication, which together are required efficient cell duplication^36–38^. Furthermore, gene set enrichment analysis demonstrated that CD271^+^ melanoma cells displayed significant correlation with high enrichment scores for the pathways containing E2F motifs and target genes. In drastic contrast, CD271^+^ cells derived from normal melanocytes displayed opposite, negative correlation scores with the same transcriptional targets (Fig. 3).

Long-term stem-like maintenance of tumor-initiating subpopulations requires efficient and active DNA repair program^39^. The differential analysis filtered for processes driven by CD271 in tumor-initiating cells revealed enrichment for the genes participating in nucleotide excision repair (NER) (Fig. 3C). This molecular pathway is of a particular importance to the developing melanomas to compensate for an excessive genomic DNA damage induced by ultraviolet (UV) light that may cause replication fork stalling and activation of the cell death machinery^40^. Activity of the nucleotide excision repair components is therefore critical to remove damaged nucleotides that would otherwise prevent effective DNA replication and cell duplication^39,40^.

Uncontrolled tumor growth is closely connected with a misbalance between cell proliferation and cell death/differentiation^41^. In addition to self-renewing factors studied in our systematic analysis of gene expression in CD271^+^ melanoma-initiating cells, a significant upregulation of the major pro-survival network controlled by AKT3 was uncovered (Fig. 5). This also included upregulation of *SHC2/4*, *GAB1* and *PI3K* genes, as well as, other critical components of the neurotrophin/CD271 signal transduction network mediating pro-survival program (Fig. 5). Interestingly, previous studies in metastatic brain melanomas had shown that CD271 knockdown causes decreased expression of AKT3 and predisposes them to the induction of apoptosis^24^. Moreover, lineage tracing of melanoma cancer stem cells exposed to a transient TNF signal showed that these cells expanded and increased in stemness in response to the TNF cue and that the PI3K/AKT pathway was necessary for this expansion^42^. Even more intriguing were recent discoveries that TNF produced in an inflammatory response by transfused engineered CD8^+^ cells in adoptive cell therapy (ACT), induces dedifferentiation and survival of CD271^+^ melanoma cells with concomitant downregulation of targeted markers, resulting in relapse and eventual disease progression^18,19^. Importantly, a recent study indicates that CD271 can be used as a guide to target tumor-initiating cells in melanoma patient derived xenografts resulting in significant suppression of metastases to lymph nodes and distant organs^17^. Moreover, *CD271* knockdown can abrogate proliferation of cells derived from melanoma as well as head and neck cancer patients to significantly reduce their tumor forming capacity in-vivo^11,26^.

In summary, by contrasting melanoma pathogenesis with normal cellular development in melanocytes, CD271 was revealed as a molecular switch. Our data demonstrate that CD271 is associated with activation and repression of distinct gene sets, which under pathological conditions control self-renewal and survival, as opposed to differentiation and cell death in the normal tissue homeostasis (Figs 1–3). The direction of the response in each case was determined by the underlying cellular fate of the specimens. In melanoma, CD271 governs processes underlying tumor progression, while in a melanocyte environment CD271 accompanies skin differentiation processes. By playing a critical role in activating signaling cascades that support melanoma cell survival and self-renewing capacity, as well as, their metastases, CD271 unveils itself as a potentially powerful therapeutic target for the treatment of metastatic disease. Further development of tumor-specific CD271 blocking modalities, including small molecule compounds that inhibit its function and subsequent activation of the downstream signal transduction pathways, identified in this report, can provide effective anti-melanoma targeting therapy approaches and synergize with immune-modulating agents.

## Materials and Methods

### Tumor cell isolation

Human tumor specimens were provided by Stanford University Hospital after obtaining informed patient consent under the Institutional Review Board Protocol (IRB) approved by Research Compliance Office of Stanford Cancer Center. Handling and isolation of human tumor cells from surgical samples was carried out in accordance with the guidelines and regulations of the approved protocol and previously described^9^. Normal, adult human melanocyte cells were a kind gift by the Dr. Ganesan laboratory, UC Irvine (originally purchased from Promocell C-12403). Briefly, tumor tissues were finely minced and incubated in Media 199 with added liberase Blendzyme TM mix at the final concentration 60 μg/ml at 37 °C. Tumor cell solution was filtered through 70 μm nylon mesh and 30 ml of HBSS containing 2 percent heat-inactivated fetal bovine serum (FBS) was added to neutralize enzyme activity, which was then centrifuged at 258 × g for 5 min at 5 °C. After several washes in HBSS containing 2 percent FBS, cells were centrifuged and resuspended in 500 μl of HBSS containing 2 percent FBS and used for the staining protocol described below.

### FACS-based purification of CD271^+^ and CD271^−^ cell populations

Prior to antibody staining, blocking reagent, mouse IgG (1 mg/ml) was added to the melanoma or adult melanocyte cell suspension and incubated on ice for 10 min. All stainings were performed in 100 μl volume of cold HBSS containing 2 percent FBS. The following lineage antibodies were added: CD45, CD31, CD2, CD3, glycophorin A, EpCAM (all conjugated to pacific blue) plus an antibody against CD271 (Alexa Fluor647-conjugated) at a 1:50 dilution. Cells were incubated on ice in the dark for 30 min. After washing and centrifugation, cells were resuspended in 0.5 ml Hank’s balanced salt solution (HBSS) containing 2% FBS and propidium iodide to allow exclusion of nonviable cells. CD271^+^ and CD271^−^ cell populations were gated based on the isotype control background signal. Isolation of CD271^+^ and CD271^−^ cells was achieved using a FACSAria III (BD Biosciences, San Jose, CA) cell sorter instrument.

### Transcriptome profiling of CD271^+^ and CD271^−^ cell populations in melanoma and melanocytes

Total RNA was extracted from CD271^+^ and CD271^−^ cell populations using Trizol Reagent (T9424-Millipore-Sigma). All RNA samples were processed using Ovation Pico WTA system V2.0 (NuGEN Technologies San Carlos, CA) and hybridized to the Human Genome U133 Plus 2.0 microarray (Affymetrix, Santa Clara, CA) chips. Following hybridization and scanning fluorescent signals were obtained for 54675 probes that were further processed and mapped to 20535 gene-coding transcripts. The RMA oligo package was used for normalization and background correction of transcriptomic data. We utilized melanoma and melanocyte specimens sorted for CD271 expression status and subjected the data to unsupervised clustering analysis. CD271^+^ and CD271^−^ specimens are denoted as CD271^+^ and CD271^−^, respectively. Both, rows (genes) and columns (specimens) were clustered using Pearson correlation distance and average linkage of log-transformed, normalized transcriptome array values. Pathway and enrichment analysis was performed by Ingenuity Pathway Analysis (IPA) software (QIAGEN Bioinformatics, Germany) and mapped with Kyoto Encyclopedia of Genes and Genomes (KEGG) pathway analysis. The enrichment study includes normalization of the enrichment score accounting for size of each gene set, yielding normalized enrichment score (NES). In addition to the calculated probability (*p* value) of hypothesis testing, there is adjustment for multiple hypothesis testing by controlling the proportion of false positives by calculating the false discovery rate (*q* values) corresponding to each NES, by comparing tails of the observed and the null distribution for the NES.

### Transcriptomic validation by qRT-PCR (quantitative real-time PCR)

Total RNA was extracted from CD271^+^ and CD271^−^ melanoma and melanocyte cells using Trizol (T9424-100ML, Millipore-Sigma, Darmstadt, Germany). The messenger RNAs of biological triplicates (N = 3) were reverse transcribed into cDNAs using Verso cDNA synthesis kits (AB1453A, Life Technologies, Carlsbad, CA), followed by real-time PCR using KAPA SYBR® FAST qPCR Master Mix (2X) Kit (Millipore-Sigma, Darmstadt, Germany) and gene-specific primer sets (Supplementary Table 2). Values were normalized against 18*S* RNA using the ΔCT method^43^,^44^.

## Supplementary information

Supplementary info

## Data Availability

Data is deposited under accession NCBI GEO entry GSE130244.
