# Supplementary material for: CD271 is a molecular switch with divergent roles in melanoma and melanocyte development
Source: Sci Rep. 2019 May 22;9:7696. doi: 10.1038/s41598-019-42773-y (PMC6531451; doi:10.1038/s41598-019-42773-y)
Supplement: Supplementary file 1 — Supplementary info [file 41598_2019_42773_MOESM1_ESM.pdf]

CD271 is a molecular switch with divergent roles in melanoma and melanocyte development

Supplementary information

Fabian V. Filipp [1, 2, 3 *], Chen Li [4,5], Alexander D. Boiko [4,5 *]

[1] Cancer Systems Biology, Institute of Computational Biology, Helmholtz Zentrum München, Ingolstädter Landstraße 1, D-85764, München, Germany.

[2] School of Life Sciences Weihenstephan, Technical University München, Maximus-von-Imhof-Forum 3, D-85354, Freising, Germany.

[3] Systems Biology and Cancer Metabolism, Program for Quantitative Systems Biology, University of California Merced, 5200 North Lake Road, Merced, CA, 95343, USA.

[4] Sue and Bill Gross Research Stem Cell Center, University of California Irvine, 845 Health Sciences Road, Irvine, CA, 92697, USA.

[5] Department of Molecular Biology and Biochemistry, University of California Irvine, 845 Health Sciences Road, Irvine, CA, 92697, USA.

[*] Correspondence: Fabian V. Filipp fabian.filipp@helmholtz-muenchen.de, Alexander D.Boiko aboiko@uci.edu

ORCID DOI: https://doi.org/10.1038/s41598-019-42773-y

Supplementary table 1: Melanoma patient samples and their characteristics

Patient ID	Diagnosis and site/Stage	*BRAF*(V600) status
**MEL213**	Regional nodal disease; Stage III	WT[1,2]
**MEL11409**	Nevoid melanoma, SQ satellite/in-transit metastasis; Stage III	Mutation[1]
**MEL425**	Dermal melanoma; Stage III	Mutation[1]
**MEL826**	Dermal melanoma; Stage IIB	Mutation[1,2]
**MEL1129**	SQ metastatic melanoma; Stage III	[ND]
**MEL1190**	SQ metastatic melanoma; Stage IV	[ND]
**MCYT**	Normal human epidermal melanocytes	[ND]

BRAF(V600) genotype verified by [1]high-resolution melt curve analysis (HRMA) or [2]Sanger sequencing. [ND] = not determined.

Supplementary table 2: Oligonucleotides primers used in this study

Gene target	Method	forward (5' → 3')	reverse (3' → 5')
18S	qRT-PCR	gcccgaagcgtttactttga	tccattattcctagctgcggt
CD271	qRT-PCR	cttctgggggtgtccctt	actcaccgctgtgtgtgta
AKT3	qRT-PCR	tggaggccaagatacttcct	gtttggctttggtcgttctg
HDAC9	qRT-PCR	tcaggatgatgatgcccgtg	tggatttgttgctgctgctg
TCF12	qRT-PCR	aatccccagcaacaacgcat	tggggaaaacatcgcactga
LEF1	qRT-PCR	cccgaagaggaaggcgattt	tggccgggatgatttcagac
